# Countdown to lobectomy: interventions to improve waiting times for lung cancer resection

**DOI:** 10.1093/intqhc/mzag035

**Published:** 2026-03-15

**Authors:** Donovan Campbell, Glenn H M Calvert, Peter Mhandu, Rory Beattie

**Affiliations:** School of Medicine, Dentistry, and Biomedical Sciences, Queen’s University Belfast, Belfast, United Kingdom; School of Medicine, Dentistry, and Biomedical Sciences, Queen’s University Belfast, Belfast, United Kingdom; Department of Cardiothoracic Surgery, Royal Victoria Hospital, Belfast, United Kingdom; Department of Cardiothoracic Surgery, Royal Victoria Hospital, Belfast, United Kingdom

## Abstract

**Background:**

Prompt diagnosis and treatment are critical to improving outcomes in patients with lung cancer. In Northern Ireland, cancer waiting times are among the worst in the UK. Since 2022, national targets have mandated that definitive treatment should commence within 62 days of referral and within 31 days of the decision to treat (DTT). The present study aimed to evaluate adherence to these standards and to assess the impact of targeted, cost-neutral interventions on waiting times.

**Methods:**

We conducted a three-cycle, prospective quality improvement study of patients undergoing lung lobectomy for primary lung cancer at a regional thoracic centre (*N *= 86). Interventions included the implementation of a joint thoracic surgery–oncology clinic and a patient pooling initiative (cycles 1–2), followed by broader service reorganization (cycles 2–3). Primary outcomes were waiting times relative to the 62- and 31-day targets; secondary outcomes were waiting times for preoperative investigations. A post-Lasso regression was performed to identify which intervals contributed most to overall (62-day) delay.

**Results:**

Following the introduction of joint clinics and patient pooling, the mean time from referral to lobectomy decreased by 27% (from 150.47 to 109.67 days; *P *= .005, *d_R_* = 0.55). No further improvement was observed following service reorganization. There was no change in time from DTT to surgery (all *P* > .05). Overall, only 5.8% and 16.3% of patients met the 62- and 31-day targets, respectively. Post-Lasso regression identified time to PET and biopsy, as well as delays between DTT, outpatient review, and lobectomy, as significant contributors to overall wait.

**Conclusions:**

Joint clinics and patient pooling initiatives were associated with significant reductions in surgical waiting times; however, compliance with national targets remained poor. While the interventions trialled were ostensibly cost-neutral, further improvement is unlikely without substantive investment in diagnostic infrastructure and operative capacity.

## Introduction

Although lung cancer remains the leading cause of cancer deaths throughout the United Kingdom [1], it remains a highly treatable disease. Its associated morbidity and mortality are reduced markedly by prompt diagnosis and treatment [2–6]. In Northern Ireland alone, more than 1300 cases of lung cancer are diagnosed per year, with as many as 44% presenting with metastatic disease [7–9]. In seeking to optimize outcomes, an impetus has been ascribed to developing regional and national guidelines to formalize optimal treatment pathways and establish benchmarks against which local health authorities can evaluate their outcomes.

Over the past decade, the prevalence of lung cancer in Northern Ireland has risen by approximately 10% [8]. The 5-year survival sits at only 12.1%, with nearly half of patients dying within 6 months of diagnosis [7–9]. This pattern of outcomes is likely attributable to the high proportion of patients presenting with advanced disease. In patients with early disease (stage I), 5-year survival is more than 30 times as great as those with metastatic disease at presentation (52.4% vs. 1.6%) [9]. Although it is to be expected that some proportion of patients will present in later stages, it is well known that delays in diagnosis and treatment correlate with upstaging of disease, and hence poorer survival [10]. Indeed, it has been demonstrated that in patients with lung cancer, nodal progression (as detected by PET) may occur in as little as 6 weeks [11].

In recognition of these challenges, a national cancer strategy was devised by the Department of Health in 2022 [7]. This framework established formal targets for waiting times that apply to all patients in whom a diagnosis of cancer is suspected. These targets stipulate that: (i) 95% of patients must be assessed by a relevant specialist within 14 days of referral; (ii) 98% of patients must commence ‘definitive’ treatment within 62 days of the initial referral; and (iii) 98% of patients must begin ‘definitive’ treatment within 31 days of the decision to treat (DTT). In the present context, i.e. lung cancers amenable to surgical intervention, ‘definitive’ treatment refers to any form of surgical resection, while the DTT corresponds to the date on which surgical intervention was recommended.

In seeking to ameliorate cancer waiting times, several strategies have been proposed. One strategy is the implementation of joint clinics, whereby the patient consults an oncologist and surgeon in the same hospital visit. In one centre, this approach—termed the ‘One Stop Lung Cancer Clinic’, engendered a five-fold decrease in median waiting times between referral and DTT [12]. It has also been shown to confer a tangible survival benefit [13]. Another strategy is patient ‘pooling’. In brief, this approach entails scheduling patients to attend the next available appointment, regardless of whether this takes place with the patient’s named consultant. This method has been shown to increase theatre efficiency—by plugging gaps arising from case cancellations, as well as reduce waiting times for elective procedures [14, 15].

The purpose of the present project was two-fold. In the first instance, we sought to assess adherence to nationally mandated waiting time targets in patients with primary lung cancer amenable to lung lobectomy. The second was to evaluate the impact of targeted, service-level interventions—namely operative and outpatient pooling, as well as broader service reorganization—on patient waiting times. Therefore, we conducted a three-cycle prospective quality improvement study of patients treated at a regional thoracic centre. To identify significant contributors to delay which may inform the basis of future interventions, a covariate-adjusted post-Lasso regression was performed on the pooled dataset.

## Materials and methods

### Interventions

A series of targeted, system-level interventions were implemented over the course of three audit cycles. The first intervention involved the introduction of a joint thoracic surgery-oncology clinic to streamline patient assessment. This approach replaced a sequential model in which patients attended separate specialists (clinical oncology, thoracic surgery) in uncoupled visits. By combining these consultations, we sought to reduce the time required for inter-specialty handover.

As a further measure, we also introduced a patient pooling initiative in both the outpatient and operative settings. This ensured that patients were booked into the next available clinic slot and next available surgical list, regardless of consultant. This method of pooling aimed to reduce variations in waiting times engendered by uneven caseloads and to maximize clinic and theatre capacity.

In the second intervention, we focused on restructuring the weekly multidisciplinary meeting (MDM). This included mandating the presence of a minimum of two thoracic surgeons at each MDM discussion, triaging cases to ensure complete staging information prior to discussion (where possible), prioritizing new and decision-ready cases, and documenting treatment decisions and action points contemporaneously to facilitate downstream scheduling.

Both intervention cycles were designed to be implemented without additional financial investment or infrastructure expansion and were considered feasible within the context of existing staff and service constraints, although they necessarily incurred opportunity costs through redeployment of existing clinical time. The lung cancer diagnostic and treatment pathway, audit cycles, and timing of service-level interventions are illustrated in Supplementary Fig. S1. Audit cycles were defined prospectively based on consecutive patient inclusion rather than fixed calendar duration.

### Study of the interventions

Interventions were introduced iteratively across defined audit cycles. Cycle lengths were based on prospectively defined patient volumes (samples of *n *= 30), rather than a fixed duration of time, to account for temporal fluctuations in operative load. Adherence was monitored through regular team meetings and by review of clinic records. Intervention effects were appraised by comparing waiting times between cycles using pre-defined outcomes and *a priori* analyses with appropriate covariate adjustments. Re-audit was undertaken following completion of each audit cycle, allowing comparison of pathway performance before and after sequential interventions (Supplementary Fig. S1).

### Outcome measures

The primary outcomes were based on patient waiting times (expressed in days) with respect to the 62-day and 31-day national cancer pathway targets. The ‘62-day wait’ was defined as the interval between the date of initial suspected cancer referral (typically from a general practitioner) to the date of lung lobectomy. The ‘31-day wait’ was defined as the interval between the date on which surgical intervention was recommended (i.e. the MDM at which the DTT was made) to the date of lung lobectomy. Waiting times were expressed continuously based on robust measures of central tendency (20% trimmed means), as well in terms of binary compliance (i.e. whether a patient was seen within the target period). This dual approach facilitated quantitative assessment of pathway efficiency, as well as compliance with percentage-based national targets.

Secondary measures included time intervals for key components of the diagnostic and preoperative pathways, namely: (i) time from referral to outpatient review, (ii) time from referral to diagnostic imaging (CT, PET), and (iii) time from referral to preoperative biopsy. These measures were obtained to identify upstream contributors to treatment delay which may inform the basis of future interventions.

All data were extracted from patient electronic care records using timestamped entries for referral, MDM discussion, diagnostic investigations, outpatient review, and operative events.

### Analysis

All statistical analyses were undertaken in R (version 4.3.3; R Foundation for Statistical Computing, Vienna, Austria) and RStudio (version 2023.12.1 + 402; RStudio, PBC, Boston, MA, USA). On initial assessment using the Shapiro–Wilk test, each distribution of waiting times failed to satisfy the conventional criterion of asymptotic normality (all *P* < .05). In seeking to minimize the potential influence of outliers and deviations from normality, robust methods were therefore employed.

Measures of central tendency were obtained using 20% trimmed means (mean_tr_), while those of dispersion were based on 20% Winsorised standard deviations (SD_w_). To evaluate the effects of interventions made across cycles, a robust two-factor analysis of variance (ANOVA) was performed with Trust (*k *= 5) and Cycle (*k *= 3) as fixed effects, using the WRS2 package in R. Based on these models, planned contrasts were undertaken with *P-*value adjusted using the Benjamini-Hochberg false discovery rate procedure. In circumstances in which a statistically significant (*P *< .05) difference was observed, effect sizes were obtained using the robust analogue of Cohen’s *d*.

To identify which pathway segments contributed most heavily to overall delay (the 62-day wait), we performed a covariate-adjusted post-Lasso regression on the pooled dataset, using the glmnet package in R. It should be emphasized that this analysis was undertaken as a method of pathway decomposition and bottleneck identification, rather than as a causal or predictive model. Given that overall waiting time represents the aggregation of component intervals, the magnitude of model fit should not be interpreted inferentially.

### Ethical considerations

This project was registered with the Belfast Health and Social Care Trust Audit Department. As a service improvement initiative based on routinely collected clinical data, there was no requirement to obtain formal research ethics approval or individual patient consent, in accordance with institutional policy. All data were anonymized prior to analysis and processed in compliance with local data protection protocols.

## Results

Overall, 86 patients were included in the study (45 females, 41 males; mean age = 67.2 years, SD = 10.5); four patients were excluded following histological analyses which revealed metastatic lesions of extrapulmonary origin. There were no significant differences in the distributions of patient sex between audit cycles [*χ^2^*(1, *N *= 86) = 0.32, *P *> .05]. Similarly, there were no significant differences in patient age between audit cycles [*F*(2, 33.65) = 1.53, *P *> .05] nor between sexes [*t*(47.53) = 1.69, *P *> .05] (Table 1).

**Table 1:** Patient age and sex stratified by audit cycle.

Cycle no.	Timeframe	*N*	Mean age (SD), years	Female *n* (%)	Male *n* (%)
1	November–December 2022	27	70.1 (9.3)	15 (55.6)	12 (44.4)
2	June–September 2023	30	65.5 (9.7)	16 (53.3)	14 (46.7)
3	September–November 2024	29	66.3 (12.1)	14 (48.3)	15 (51.7)
Total	—	86	67.2 (10.5)	45 (52.3)	41 (47.7)

**Figure 1:**
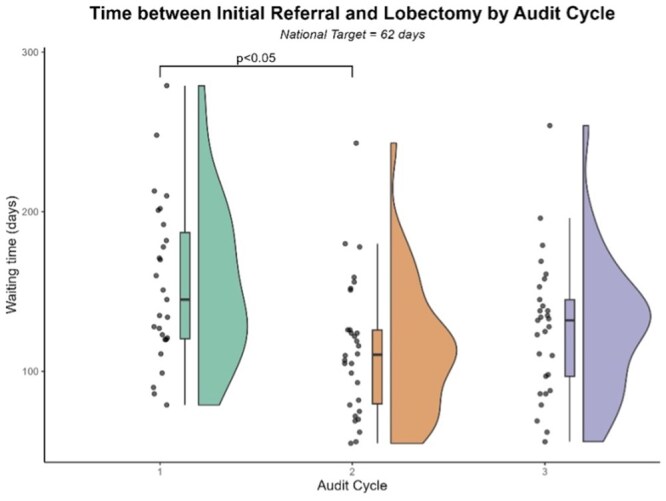
Raincloud plot illustrating waiting time between initial referral and lung lobectomy (62-day wait) as a function of cycle.

Between Cycle 1 (baseline) and Cycle 2, following the introduction of joint clinics and patient pooling initiatives, the mean interval from referral to lobectomy (the ‘62-day wait’) decreased from 150.47 days (SD_w_ = 32.84) to 109.67 days (SD_w_ = 28.80), representing a 27% relative reduction [Yuen’s *t*(31.85) = 3.01, *P *= .005, *d_R_* = 0.55]. However, no further improvement was observed in Cycle 3 following service reorganization (mean_tr_ = 124.89 days, *SD_w_* = 26.25). The difference between Cycles 1 and 3 did not achieve statistical significance (*P *> .05). A robust two-factor ANOVA demonstrated no main effect of Trust and no Cycle × Trust interaction (all *P* > .05; Fig. 1), indicating that the observed improvement was confined to a single audit cycle. In terms of binary compliance, only 5/86 (5.8%) patients underwent lobectomy within 62 days of referral (against a target of 98%) with no variation noted between cycles [*χ^2^* (2, *N *= 86) = 2.69, *P *> .05].

**Figure 2:**
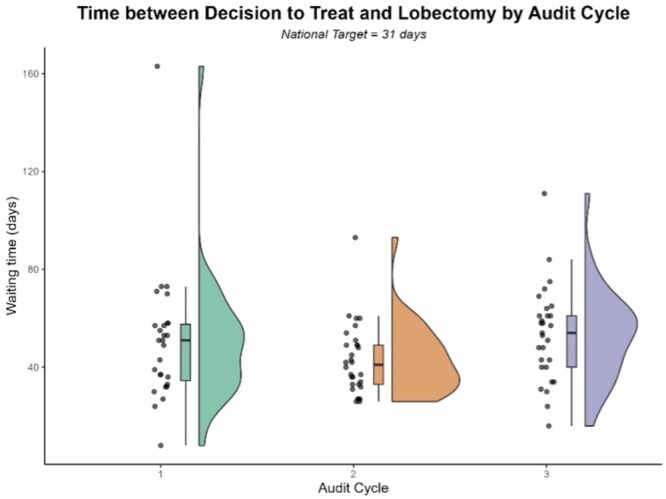
Raincloud plot illustrating waiting time between decision-to-treat and lung lobectomy (31-day wait) as a function of cycle.

With respect to 31-day waits (i.e. the interval between DTT and lobectomy), no significant differences were observed between cycles (cycle 1: mean_tr_ = 47.00 days, SD_w_ = 10.95; cycle 2: mean_tr_ = 40.89 days, SD_w_ = 8.06; cycle 3: mean_tr_ = 52.53 days, SD_w_ = 10.67; all *P* > .05). A robust two-factor ANOVA with Cycle and Trust as fixed effects revealed no effect of Cycle, Trust, or a Cycle x Trust interaction (all *P* > .05). Overall, only 14/86 (16.3%) patients underwent lobectomy within 31 days of the DTT (against a target of 98%) with no variation noted between cycles [*χ^2^* (2, *N *= 86) = 0.48, *P *> .05]. The findings are illustrated in Fig. 2.

**Figure 3:**
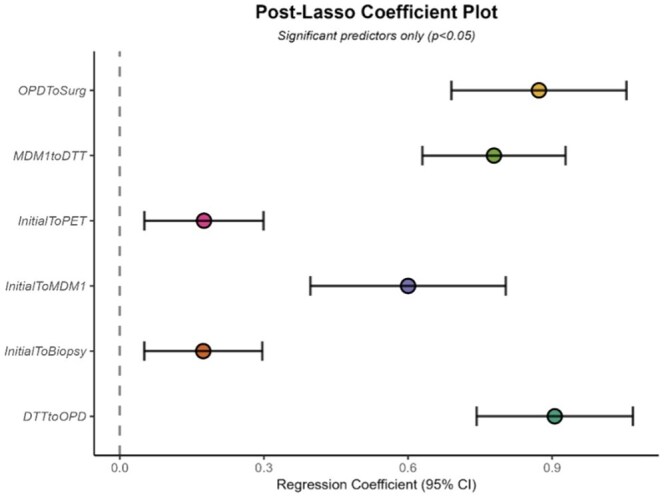
Coefficient plot of significant contributors to overall (62-day) waiting time, as derived from post-Lasso regression. OPD = outpatient department clinic appointment; MDM1 = first MDM discussion. Error bars denote 95% confidence intervals. Note that ‘Initial’ refers to the date of initial referral.

To identify which components of the lung cancer pathway contributed most strongly to overall (i.e. 62-day) waiting time, a post-Lasso regression was performed. As expected, the model explained a high proportion of variance, reflecting the aggregation of pathway intervals rather than independent prediction (*R*^2^ = 0.94). This analysis revealed that shorter waits to preoperative investigations, namely PET (*β* = 0.18) and biopsy (*β* = 0.17)—but notably not CT, were associated with shorter waits to surgery (*P* < .05). Delays between referral to first MDM discussion (*β* = 0.60), as well as from first MDM discussion to DTT (*β* = 0.78), were also associated significantly with overall wait. Waiting times between DTT and joint clinic attendance (*β* = 0.91), as well as between joint clinic and lobectomy (*β* = 0.87), were however most strongly associated with 62-day waits (see Fig. 3). No other intervals attained statistical significance.

To ascertain which components of the lung cancer pathway varied significantly between cycles, we examined mean waiting times for several diagnostic, decision-making, and post-decision intervals by cycle (Table 2). Across most pathway components, trimmed mean waiting times were numerically lower in Cycle 2 compared with baseline, with partial regression in Cycle 3. The largest absolute reduction was observed for referral-to-PET time, which decreased from 56.1 days in Cycle 1 to 39.3 days in Cycle 2 and 29.4 days in Cycle 3. Robust omnibus testing was performed for each interval, with *P*value adjusted for multiple comparisons using the false discovery rate procedure. Following adjustment, no individual pathway interval demonstrated a statistically significant between-cycle difference, and no significant between-cycle variation was observed for downstream surgical intervals or overall referral-to-surgery time. These findings provide additional descriptive context for the bottleneck analysis above (Fig. 3).

**Table 2:** Trimmed mean waiting times (days) for lung cancer pathway intervals by audit cycle.

Pathway interval	Cycle 1 (SDw)	Cycle 2 (SDw)	Cycle 3 (SDw)	Omnibus *p_FDR_*
Referral to CT^a^	−3.8 (5.3)	−7.0 (8.6)	−5.9 (9.4)	0.638
Referral to PET	56.1 (19.6)	39.3 (20.6)	29.4 (11.8)	0.072
Referral to Biopsy	80.5 (32.1)	61.9 (20.3)	55.5 (24.4)	0.472
Referral to MDM1	43.5 (17.5)	37.8 (22.3)	40.6 (18.7)	0.638
MDM1 to DTT	56.9 (32.1)	31.1 (13.8)	35.6 (25.2)	0.638
DTT to OPC	13.9 (7.4)	13.2 (4.7)	14.9 (5.6)	0.638
OPC to surgery	32.1 (8.3)	27.0 (5.6)	37.5 (11.10)	0.638
Referral to surgery	150.5 (33.4)	109.7 (28.6)	124.9 (27.4)	0.0776

pFDR = false discovery rate-adjusted *P*-value for between-cycle differences.

a Negative values indicate imaging performed prior to suspected cancer referral.

## Discussion

### Statement of principal findings

Here, we demonstrate that the introduction of joint clinics and patient pooling initiatives was associated with a significant reduction in waiting times between referral and lung cancer lobectomy (the ‘62-day’ wait). Collectively, these interventions were associated with a 27% reduction in average waiting times, reflecting significant improvement in a pathway characterized by substantial delays. No further improvements were associated with a service reorganization initiative. Similarly, no interventions were effective in reducing waiting times between DTT and lobectomy (i.e. the ‘31-day’ wait).

Despite improvements in 62-day waits, there was little variation in binary compliance, which fell far short of national targets. Indeed, fewer than 6% of patients underwent lobectomy within 62 days of referral, against a national target of 98%. Decomposition of the lung cancer pathway into its constituent intervals demonstrated that the largest numerical reductions occurred within diagnostic components, particularly referral-to-PET and referral-to-biopsy intervals, whereas downstream surgical intervals exhibited little variation between audit cycles. Delays to initial MDM discussion and to DTT were associated with longer overall waits to surgery, while faster progression from DTT through joint clinic attendance to lobectomy was most strongly associated with overall pathway duration. It should be emphasized that these findings reflect where time accumulates along the pathway and do not necessarily represent independent predictors of delay.

Importantly, the observed improvement in referral-to-lobectomy waiting time was confined to a single audit cycle and was not sustained following subsequent service reorganization. This pattern suggests that the reduction observed in Cycle 2 may reflect a combination of intervention effects and contextual factors, including short-term optimization of clinic and theatre utilization, temporal variation in case mix, or stochastic fluctuations in diagnostic and operative capacity. In the absence of a control group, and given the iterative, real-world nature of quality improvement work, causality cannot therefore be inferred. Notably, Cycle 3 did not succeed any expansion of PET or biopsy capacity, which were identified as dominant contributors to overall delay, which may explain the lack of improvement observed following the service reorganization initiative.

The identification of diagnostic investigations as dominant contributors to overall pathway delay has important implications for service redesign. In this cohort, referral-to-PET and referral-to-biopsy intervals demonstrated the greatest numeric variation across audit cycles, whereas downstream surgical intervals exhibited little change. This suggests that efforts to expand surgical capacity alone are unlikely to deliver durable improvements in waiting times without parallel optimization of diagnostic services.

Potential strategies to reduce diagnostic delays include protected PET slots for cancer referrals, increased utilization of networked PET capacity across neighbouring centres, and earlier triaging of patients for biopsy based on initial imaging and clinical risk. At a local level, this may involve formal prioritization of lung cancer cases within PET scheduling systems or closer alignment of respiratory, radiology, and pathology workflows to minimize sequential delays. More broadly, these findings support national and regional initiatives aimed at expanding access to advanced imaging and tissue diagnosis, without which improvements in downstream surgical efficiency may not translate into meaningful reductions in overall wait.

### Interpretations within the context of the wider literature

In implementing a joint thoracic surgery-oncology clinic, we show that streamlining patient assessment may reduce surgical waiting times by as much as 27%. This is consistent with the effects reported in two cohorts of patients awaiting breast cancer surgery, which reported decreases in the order of 29%–65% [16, 17]. It also builds upon the benefits conferred by ‘one stop clinics’, which have been shown previously to reduce the interval between referral and DTT in patients with lung cancer [12, 13]. By integrating appointments into a single visit, joint clinics may diminish lags in inter-specialty handover and facilitate earlier formulation of treatment plans [18, 19]. Indeed, under this model, 100% of patients with a diagnosis of lung cancer received a DTT within 30 days of referral—more than double the rate observed in standard care [12].

### Strengths and limitations

Previous studies have demonstrated that patient pooling initiatives may ameliorate waiting lists by leveraging ‘spare’ hospital capacity, e.g. by filling clinic and theatre slots vacated at short notice [14, 20]. In the present study, clinic and operative patient pooling was implemented alongside the introduction of a joint clinic model. Although it is not possible to isolate the individual effects of each intervention (as their dual implementation precludes partitioning of their variances), it is clear that at least in combination, these interventions yielded a substantial reduction in surgical waiting times. A particular advantage of pooling is that it prioritizes patient urgency rather than consultant availability, whilst buffering against short-term disruptions, e.g. illness, leave, and other causes of cancellation. Although pooling alone is unlikely to resolve systemic bottlenecks, such as limited theatre availability, our findings suggest that it represents a simple, scalable tool that may reduce inefficiencies within the context of existing resource constraints.

The present study is not without its limitations. In the first instance, the study was undertaken at a single tertiary centre, which may limit the generalizability of our findings; however, as all onco-thoracic surgery in Northern Ireland is centralized to our institution, our findings should be considered representative of regional practice. Secondly, as joint clinics and pooling initiatives were implemented concurrently, it is not possible to determine their individual effects or to model their interaction. Although these interventions are known to confer benefit when introduced independently, a sequential (rather than concurrent) implementation is required to estimate the relative magnitude of their individual effects. Thirdly, although the effect size associated with joint clinics and pooling was moderate in magnitude (*d_R_* = 0.55), the post-hoc power of our study was only 53% (below the conventional threshold of 80%). As a sample size of 53 patients per cycle would be required to replicate our findings, considerable circumspection is therefore required. Indeed, it is likewise plausible that the lack of improvement noted in Cycle 3 may reflect a Type II error due to limited statistical power. Lastly, although post-Lasso regression is ideal for variable selection in the presence of multicollinearity (and thus beneficial in identifying temporal contributors to delay), it is nonetheless the subject of inherent limitations, most notably its potential for penalization and exclusion of covariates with modest effect sizes.

An additional limitation of this study relates to its pre–post design and the absence of a contemporaneous control group. As a result, it is not possible to attribute observed changes in waiting times solely to the interventions implemented, nor to exclude the influence of secular trends, unmeasured service pressures, or external system-level factors operating over the study period. Although the iterative, real-world nature of quality improvement work necessitates such designs, these findings should be interpreted as associative rather than causal, and replication within controlled or multi-centre frameworks would be required to establish causality. A further limitation relates to potential confounding by case mix. Although waiting times in lung cancer pathways are influenced by tumour stage, physiological fitness, and the complexity of required staging investigations, we did not have access to sufficiently complete or standardized data on these variables across all audit cycles to permit meaningful adjustment. As a result, residual confounding by unmeasured patient-level factors cannot be excluded.

While the present study highlights actionable targets for pathway improvement, there remains considerable uncertainty concerning the capacity of our service to absorb any increased demand. Notably, CT was not a significant predictor of overall (i.e. 62-day) wait, which may reflect the fact that many lung cancers in Northern Ireland are detected incidentally. The more pressing constraint however lies in access to PET and biopsy services, where it is capacity rather than speed (i.e. of reporting) that is the obvious limiting factor. Importantly, our findings suggest that earlier delays in the patient journey—specifically those preceding and between MDM discussions—contributed significantly to overall wait, highlighting the need to streamline pre-MDM workup. Our experience was that delays between first MDM discussion and DTT were often due to incomplete staging investigations, underscoring the importance of timely access to PET and biopsy to enable early decision-making. In this context, collaboration with private providers or centres in the Republic of Ireland may merit exploration. Conversely, reducing delays between DTT, outpatient review, and surgery is likely to require more fundamental restructuring, such as additional outpatient clinics, increased theatre provision, and anaesthetic support. The development of elective surgical hubs, weekend operating lists, or the repurposing of surgical infrastructure elsewhere may offer partial solutions.

### Implications for policy, practice, and research

Looking ahead, the introduction of lung cancer screening in Northern Ireland offers promise in improving stage at diagnosis and expanding the pool of patients eligible for curative treatment. According to data from the UK Lung Cancer Screening Pilot, 85.7% of patients detected by lung cancer screening are diagnosed at stages I–II, compared with approximately 30% under routine care [21, 22]; approximately 83.3% of screened patients subsequently underwent surgical resection [21]. Screening has also been shown to confer significant mortality benefit, including a 35% relative reduction in a UK cohort, and a 16% reduction across nine international trials [22]. The value of screening is not however intrinsic, but rather conditional on the system’s capacity to deliver treatment to the patients thus detected. Without commensurate expansions in diagnostic and operative capacity, earlier detection may simply result in earlier queuing, thereby exacerbating pressure on an already constrained system.

## Conclusions

In conclusion, this study demonstrates that the introduction of joint thoracic surgery–oncology clinics and patient pooling initiatives was associated with a meaningful reduction in surgical waiting times for patients with resectable lung cancer. However, despite a 27% improvement in 62-day waits, waiting times continued to vastly exceed national targets. Post-Lasso regression and pathway interval analyses identified diagnostic delays, particularly access to PET imaging and timely biopsy, as key upstream bottlenecks influencing overall pathway duration, alongside delays between DTT, outpatient review, and lobectomy. Efforts to improve performance against national benchmarks should therefore prioritize optimization of diagnostic capacity and coordination, in parallel with downstream surgical processes. While the interventions trialled were intentionally cost-neutral and designed to optimize existing infrastructure (albeit with opportunity costs incurred by clinician redeployment), further gains are unlikely to be achieved without substantive investment—specifically in improving access to diagnostic services, increasing outpatient clinic capacity, and expanding operative provision through additional theatre lists or by leveraging spare surgical capacity in centres elsewhere.

## Supplementary Material

mzag035_Supplementary_Data

## Data Availability

The data underlying this article cannot be shared publicly for the privacy of data of individuals included within the study. The data will be shared on reasonable request to the corresponding author.
