# Supplementary figures and images for: Countdown to lobectomy: interventions to improve waiting times for lung cancer resection
Source: Int J Qual Health Care. 2026 Mar 15;38(1):mzag035. doi: 10.1093/intqhc/mzag035 (PMC13019523; doi:10.1093/intqhc/mzag035)

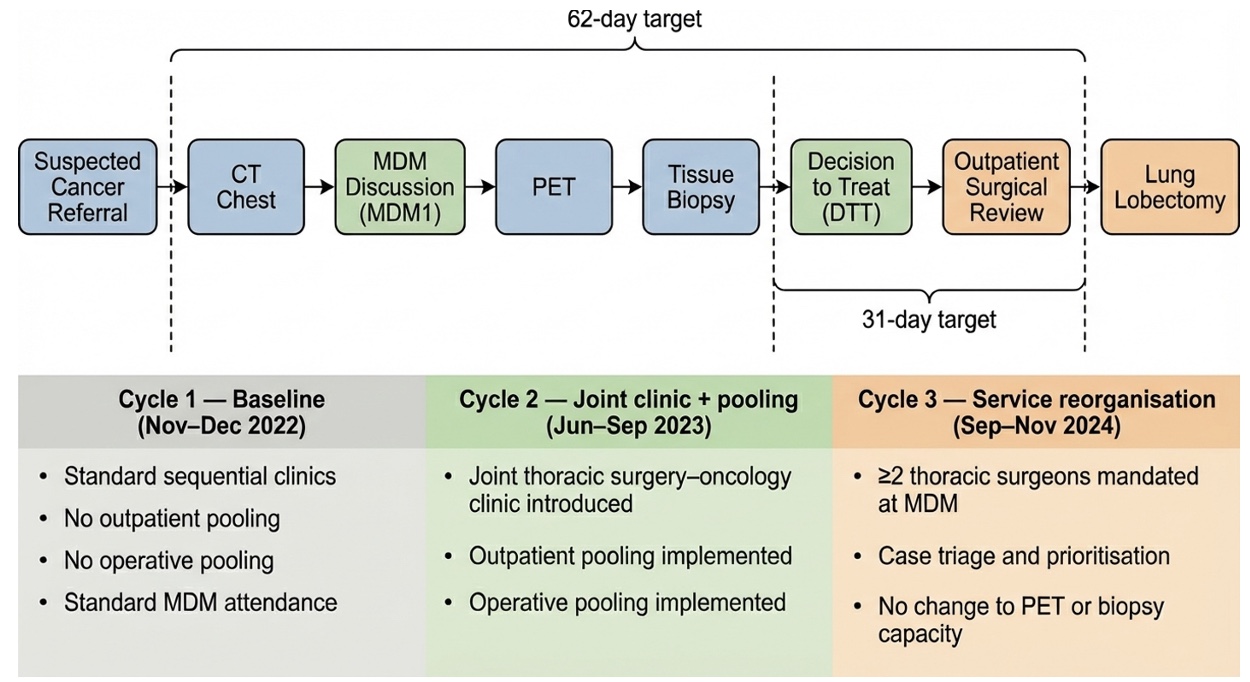

Supplement: mzag035_Supplementary_Data [file mzag035_supplementary_data.zip › Image 01-03-2026 at 17.54 (1).jpeg]
