# Supplementary material for: Countdown to lobectomy: interventions to improve waiting times for lung cancer resection
Source: Int J Qual Health Care. 2026 Mar 15;38(1):mzag035. doi: 10.1093/intqhc/mzag035 (PMC13019523; doi:10.1093/intqhc/mzag035)
Supplement: mzag035_Supplementary_Data [file mzag035_supplementary_data.zip › QIP_Revisions_Overview.docx]

Ms. No. *INTQHC-2025-07-0336*

Title: *Countdown to Lobectomy: Interventions to Improve Waiting Times for Lung Cancer Resection*

**Response to Reviewers**

We are grateful for the time and effort committed by the reviewers in critically evaluating our manuscript. Our responses to the concerns and questions of each reviewer are presented below. In the revised version of the manuscript, modified sections of text are shown in red font.

Reviewer 1

| Comments | How this was addressed |
| --- | --- |
| 1- The timeline of defining standards, intervention and re-audit are not clear. The timeline between this is of importance, especially with repeat re-audit data in your set.  ***I recommend generating a flow chart for inclusion as a figure or supplementary figure.*** | We thank the reviewer for this suggestion. We have clarified the temporal structure of the study. A pathway flow chart has been added as Supplementary Figure S1, illustrating the lung cancer diagnostic and treatment pathway, nationally mandated waiting time standards, audit cycles, and timing of service-level interventions.  In the methods section, the following **additions** have been made:  **Methods: Interventions**  *The lung cancer diagnostic and treatment pathway, audit cycles, and timing of service-level interventions are illustrated in Supplementary Figure S1. Audit cycles were defined prospectively based on consecutive patient inclusion rather than fixed calendar duration.*  **Methods: Study of the interventions**  *Re-audit was undertaken following completion of each audit cycle, allowing comparison of pathway performance before and after sequential interventions (Figure S1).* |
| 2- The claim that the 27% improvement in 61-day wait is somewhat misleading. Figure 1. demonstrates a significant difference between cycle 1 and cycle 2 but no significant difference between 2 and 3 and, importantly, between 1 and 3.  ***There may be a multitude of factors responsible for an isolated improvement during a given cycle. This should be presented with greater clarity in the results and the reasoning discussed to greater detail.*** | We have revised the Results and Discussion to clarify that the observed 27% reduction in referral-to-lobectomy waiting time was confined to Cycle 2 and was not sustained in Cycle 3. The Results now explicitly report the absence of a significant difference between Cycles 1 and 3, and the Discussion has been amended to emphasise that other factors may have contributed to an isolated improvement in a single audit cycle.  The following amendments have been made:  **Results**  The text reading:  *Following the introduction of joint clinics and pooling initiatives, the mean interval from referral to lobectomy (i.e., the “62-day wait”) decreased significantly from cycle 1 (meantr = 150.47 days, SDw = 32.84) to cycle 2 (meantr = 109.67 days, SDw = 28.80) (Yuen’s t(31.85) = 3.01, p = 0.005, dR = 0.55). No further improvements were achieved by service reorganisation as evident in cycle 3 (meantr = 124.89 days, SDw = 26.25, p > 0.05). A robust two-factor ANOVA with Cycle and Trust as fixed effects revealed no effect of Trust or a Cycle x Trust interaction (both ps > 0.05, see Figure 1).*  Has been **replaced** with:  *Between Cycle 1 (baseline) and Cycle 2, following the introduction of joint clinics and patient pooling initiatives, the mean interval from referral to lobectomy (the ‘62-day wait’) decreased from 150.47 days (SDw = 32.84) to 109.67 days (SDw = 28.80), representing a 27% relative reduction (Yuen’s t(31.85) = 3.01, p = 0.005, dR = 0.55). However, no further improvement was observed in Cycle 3 following service reorganisation (meantr = 124.89 days, SDw = 26.25). The difference between Cycles 1 and 3 did not achieve statistical significance (p > 0.05). A robust two-factor ANOVA demonstrated no main effect of Trust and no Cycle × Trust interaction (all ps > 0.05; Figure 1), indicating that the observed improvement was confined to a single audit cycle.*  **Discussion: Summary of principal findings**  An additional paragraph has been **added** which reads:  *Importantly, the observed improvement in referral-to-lobectomy waiting time was confined to a single audit cycle and was not sustained following subsequent service reorganisation. This pattern suggests that the reduction observed in Cycle 2 may reflect a combination of intervention effects and contextual factors, including short-term optimisation of clinic and theatre utilisation, temporal variation in case mix, or stochastic fluctuations in diagnostic and operative capacity. In the absence of a control group, and given the iterative, real-world nature of quality improvement work, causality cannot therefore be inferred.* *Notably, Cycle 3 did not succeed any expansion of PET or biopsy capacity, which were identified as dominant contributors to overall delay, which may explain the lack of improvement observed following the service reorganisation initiative.* |
| 3- Many of the factors explored in Figure 3 overlap and i question the relevance of them alone without introducing mean times per item, per audit cycle - perhaps as a table.  ***The table should give mean times +/- SD for period 1/2/3 and test for significant differences between time points.***  This is an important component - at present i don't believe the authors have sufficiently explored the results of their intervention and the possible contributors to same. | We agree that examining individual pathway intervals is important for understanding contributors to overall delay. Accordingly, we added a pathway interval analysis reporting mean waiting times by audit cycle, alongside robust omnibus testing for between-cycle differences. Given the number of intervals examined and the modest sample size within each cycle, we limited inferential testing to omnibus comparisons and did not perform routine post hoc pairwise testing, in order to avoid an inflated multiple-comparisons burden and low statistical power. A table has been added following the reviewer’s suggestion (see Supplementary section).  **Results**  The following text has been **added**:  *To ascertain which components of the lung cancer pathway varied significantly between cycles, we examined mean waiting times for several diagnostic, decision-making, and post-decision intervals by cycle (Table 2). Across most pathway components, trimmed mean waiting times were numerically lower in Cycle 2 compared with baseline, with partial regression in Cycle 3. The largest absolute reduction was observed for referral-to-PET time, which decreased from 56.1 days in Cycle 1 to 39.3 days in Cycle 2 and 29.4 days in Cycle 3. Robust omnibus testing was performed for each interval, with p-values adjusted for multiple comparisons using the false discovery rate procedure. Following adjustment, no individual pathway interval demonstrated a statistically significant between-cycle difference, and no significant between-cycle variation was observed for downstream surgical intervals or overall referral-to-surgery time. These findings provide additional descriptive context for the bottleneck analysis above (Figure 3).*  **Discussion**  The text reading:  *Notably, shorter waits for preoperative investigations, namely biopsy and PET (but not CT), were associated with favourable waits to surgery. By contrast, delays to initial MDM discussion – and thereafter DTT, were associated with longer waits to surgery. Faster turnarounds between DTT and joint clinic attendance, followed by lobectomy, were however the strongest predictors of overall (i.e., 62-day) wait.*  Has been **replaced** with:  *Decomposition of the lung cancer pathway into its constituent intervals demonstrated that the largest numerical reductions occurred within diagnostic components, particularly referral-to-PET and referral-to-biopsy intervals, whereas downstream surgical intervals showed little variation across audit cycles. Delays to initial multidisciplinary discussion and to DTT were associated with longer overall waits to surgery, while faster progression from DTT through joint clinic attendance to lobectomy remained the strongest predictor of overall pathway duration.* |
| 4- The discussion and conclusion are somewhat contradictory to the results.  ***The main finding of this study is identification of a bottleneck at PET and at biopsy which influences overall wait-time.***  However, the discussion focuses on increasing surgical capacity in sections and does not sufficiently elaborate on modes of PET and biopsy time improvement that are possible/ suitable to their centre and/or generalisable to the field.  This is essential and should be re-evaluated. | We agree with the reviewer that the principal finding of this study is the identification of diagnostic investigations, particularly PET imaging and biopsy, as dominant bottlenecks influencing overall pathway duration. We have therefore revised both the Discussion and the Conclusion to more clearly align with this finding.  In the discussion, the following **additions** were made:  **Discussion: Summary of principal findings**  *The identification of diagnostic investigations as dominant contributors to overall pathway delay has important implications for service redesign. In this cohort, referral-to-PET and referral-to-biopsy intervals demonstrated the greatest numerical variation across audit cycles, whereas downstream surgical intervals showed little change. This suggests that efforts to expand surgical capacity alone are unlikely to deliver durable improvements in pathway-wide waiting times in the absence of parallel optimisation of diagnostic services.*  *Potential strategies to reduce diagnostic delays include protected PET slots for cancer referrals, increased utilisation of networked PET capacity across neighbouring centres, and earlier triaging of patients for biopsy based on initial imaging and clinical risk. At a local level, this may involve formal prioritisation of lung cancer cases within PET scheduling systems or closer alignment of respiratory, radiology, and pathology workflows to minimise sequential delays. More broadly, these findings support national and regional initiatives aimed at expanding access to advanced imaging and tissue diagnosis, without which improvements in downstream surgical efficiency may fail to translate into meaningful reductions in overall pathway duration.*  In the conclusion, the following **amendments** were made:  **Discussion: Conclusion**  *In conclusion, this study demonstrates that the introduction of joint thoracic surgery–oncology clinics and patient pooling initiatives was associated with a meaningful reduction in surgical waiting times for patients with resectable lung cancer. However, despite a 27% improvement in 62-day waits, waiting times continued to vastly exceed national targets. Post-Lasso regression and pathway interval analyses identified diagnostic delays, particularly access to PET imaging and timely biopsy, as key upstream bottlenecks influencing overall pathway duration, alongside delays between DTT, outpatient review, and lobectomy. Efforts to improve performance against national benchmarks should therefore prioritise optimisation of diagnostic capacity and coordination, in parallel with downstream surgical processes. While the interventions trialled were intentionally cost-neutral and designed to optimise existing infrastructure (albeit with opportunity costs incurred by clinician redeployment), further gains are unlikely to be achieved without substantive investment – specifically in improving access to diagnostic services, increasing outpatient clinic capacity, and expanding operative provision through additional theatre lists or by leveraging spare surgical capacity in centres elsewhere.* |

Reviewer 2

| Comments | How this was addressed |  |
| --- | --- | --- |
| 1- Circularity in the Regression  The regression model predicts the 62-day wait using its component intervals (e.g. referral→MDM, DTT→surgery).  Because the outcome is essentially the sum of these intervals, it is inevitable that the model will explain most of the variance, resulting in a very high R².  The analysis does not identify independent predictors of delay, but rather highlights where time accumulates along the pathway.  ***You could do a bottleneck analysis showing where the delays occur or restrict predictors to patient- or system-level characteristics (e.g. stage, fitness, comorbidity, diagnostic modality) that might explain why some patients experience longer waits than others?*** | We thank the reviewer for this important and well-taken methodological observation. We agree that modelling overall (62-day) waiting time using its component pathway intervals introduces an inherent degree of circularity, and that this necessarily inflates the proportion of explained variance.  Our intention in performing the post-Lasso regression was not to identify independent causal predictors of delay, but rather to decompose the lung cancer pathway and identify where time preferentially accumulates, thereby highlighting operational bottlenecks most amenable to service-level intervention. In this context, the analysis is best interpreted as a structured bottleneck analysis rather than a predictive or explanatory model.  We have revised the manuscript to make this explicit. The following amendments have therefore been made:  **Methods: Analysis**  The text which read:  *To identify factors predictive of overall waiting time (i.e., the “62-day wait”), a covariate-adjusted post-Lasso regression was performed on the pooled dataset, using the glmnet package in R. The benefit of this approach is that it facilitates variable selection and regularisation in the presence of multi-collinearity, while also ameliorating the risk of overfitting. This method yields interpretable, unbiased coefficient estimates whose magnitudes may inform the targets of future interventions.*  Has been **replaced** with:  *To identify which pathway segments contributed most heavily to overall delay (the 62-day wait), we performed a covariate-adjusted post-Lasso regression on the pooled dataset, using the glmnet package in R. It should be emphasised that this analysis was undertaken as a method of pathway decomposition and bottleneck identification, rather than as a causal or predictive model. Given that overall waiting time represents the aggregation of component intervals, the magnitude of model fit should not be interpreted inferentially.*  This has been reinforced in the **results** section with the following amendment:  *To identify which components of the lung cancer pathway contributed most strongly to overall (i.e. 62-day) waiting time, a post-Lasso regression was performed. As expected, the model explained a high proportion of variance, reflecting the aggregation of pathway intervals rather than independent prediction (R2 = 0.94).*  In addition, we have added the following sentence to the **discussion** section when reporting these findings:  *It should be emphasised that these findings reflect where time accumulates along the pathway and do not necessarily represent independent predictors of delay.*  While we agree that alternative modelling approaches using patient- or system-level characteristics (e.g., tumour stage, physiological fitness, diagnostic modality) could help explain why some patients experience longer waits than others, we do not unfortunately have access to these data.  To highlight this limitation to the reader, the following text has been added to the strengths and limitations section of the **discussion**:  *Although waiting times in lung cancer pathways are influenced by tumour stage, physiological fitness, and the complexity of required staging investigations, we did not have access to sufficiently complete or standardised data on these variables across all audit cycles to permit meaningful adjustment. As a result, residual confounding by unmeasured patient-level factors cannot be excluded.* |  |
| 2- Insufficient detail on Cycle 3 (“service reorganisation”) Cycle 3 is vague.  It is difficult to interpret why no further improvement was observed.  ***Provide description of service reorganisation*** | We thank the reviewer for highlighting the need for greater clarity regarding the nature of the Cycle 3 intervention. We agree that the term “service reorganisation” was insufficiently specific in the original manuscript.  In the revised version, we now explicitly describe the Cycle 3 intervention as a restructuring of the multidisciplinary decision-making process, including mandating the presence of a minimum of two thoracic surgeons at each lung cancer MDM and streamlining case discussion workflows.  The following amendments have therefore been made:  **Methods: Interventions**  The text which read:  *In the second intervention, we focused on restructuring the weekly multidisciplinary meeting (MDM). To prevent delays in decision-making and to improve continuity of care, we mandated that a minimum of two thoracic surgeons must be present at each MDM. This helped to ameliorate delays arising from the absence of senior decision-makers.*  Has been **replaced** with:  *In the second intervention, we focused on restructuring the weekly multidisciplinary meeting (MDM). This included mandating the presence of a minimum of two thoracic surgeons, triaging cases to ensure complete staging information prior to discussion where possible, prioritising new and decision-ready cases, and documenting treatment decisions and action points contemporaneously to facilitate downstream scheduling.* | DC |
| 3- Causal language Phrases such as “engendered” or “demonstrates that” imply causality.  This can't be supported by a pre–post design and no control group.  ***Amend*** | We thank the reviewer for highlighting this important point. We agree that causal language is not appropriate in the context of a pre–post quality improvement study without a control group.  In the revised manuscript, we have therefore amended causal phrasing throughout (e.g. “engendered”) to more neutral associative language (e.g. “was associated with”, “coincided with”). We have also reinforced the non-causal interpretation of our findings in the Discussion. These changes do not alter the results presented, but more accurately reflect the study design.  Throughout the manuscript, causal language has been removed and substituted (changes highlighted in red).  In addition, we have added a further limitation to our **discussion** section which reads:  *An additional limitation of this study relates to its pre–post design and the absence of a contemporaneous control group. As a result, it is not possible to attribute observed changes in waiting times solely to the interventions implemented, nor to exclude the influence of secular trends, unmeasured service pressures, or external system-level factors operating over the study period. Although the iterative, real-world nature of quality improvement work necessitates such designs, these findings should be interpreted as associative rather than causal, and replication within controlled or multi-centre frameworks would be required to establish causality.* | DC |
| 4- Confounding/case mix  Table 1 reports age and sex only. In lung cancer pathways, delays are strongly influenced by tumour stage, physiological fitness, and need for complex staging investigations.  ***Should consider including at least some basic clinical characteristics across cycles.*** | We thank the reviewer for highlighting the potential influence of case mix on pathway waiting times. We agree that tumour stage, physiological fitness, and the need for complex staging investigations are important determinants of diagnostic and treatment timelines in lung cancer pathways. Unfortunately, we did not have access to sufficiently complete or standardised data on tumour stage, performance status, comorbidity burden, or staging complexity across all audit cycles to permit reliable adjustment or stratification by these variables. As such, age and sex were the only patient-level characteristics available for consistent comparison between cycles.  We have acknowledged this explicitly as a limitation in the revised manuscript. However, we note that the primary objective of this quality improvement study was to examine system-level pathway performance over time, rather than to model inter-patient heterogeneity. To this end, our analyses focused on pathway intervals and service-level interventions, which are less sensitive to individual patient characteristics than outcome-based measures. Nonetheless, we agree that residual confounding by case mix cannot be excluded, and that future studies incorporating clinical staging and fitness data would be valuable.  The following text has therefore been added to the limitations section of our **discussion**.  *A further limitation relates to potential confounding by case mix. Although waiting times in lung cancer pathways are influenced by tumour stage, physiological fitness, and the complexity of required staging investigations, we did not have access to sufficiently complete or standardised data on these variables across all audit cycles to permit meaningful adjustment. As a result, residual confounding by unmeasured patient-level factors cannot be excluded.* | DC |
| 5- ‘Cost-neutral’ claim  While no new financial investment was made, mandating two surgeon presence at MDMs and restructuring clinics does carry opportunity costs.  ***Amend*** | We thank the reviewer for this important clarification. We agree that, although no additional financial investment or infrastructure was introduced as part of the interventions, the reorganisation of multidisciplinary meetings and outpatient workflows necessarily incurred opportunity costs, including redeployment of existing clinical time.  We have amended the manuscript to reflect this distinction more clearly.  **Methods: Interventions**  The text which read:  *Both intervention cycles were designed to be cost-neutral and feasible within the context of existing staff and infrastructure constraints.*  Has been **amended** to:  *Both intervention cycles were designed to be implemented without additional financial investment or infrastructure expansion and were feasible within the context of existing staff and service constraints, although they necessarily incurred opportunity costs through redeployment of existing clinical time.*  Similarly, we have **modified** the **conclusion** with the following:  *While the interventions trialled were intentionally cost-neutral and designed to optimise existing infrastructure (albeit with opportunity costs incurred by clinician redeployment), further gains are unlikely to be achieved without substantive investment – specifically in improving access to diagnostic services, increasing outpatient clinic capacity, and expanding operative provision through additional theatre lists or by leveraging spare surgical capacity in centres elsewhere.* | DC |
| 6- Sample size  The authors appropriately acknowledge limited power.  This is particularly true in Cycle 3 which may reflect a Type II error. | We agree with the reviewer that the modest sample size, particularly within Cycle 3, raises the possibility of a Type II error. We have addressed this by adding an explicit statement to the strengths and limitations section of the **discussion,** noting that the absence of improvement observed in Cycle 3 may reflect limited statistical power.  Specifically, the following sentence has been **added** to the **discussion** following our discussion of post-hoc power.  *Indeed, it is likewise plausible that the lack of improvement noted in Cycle 3 may reflect a Type II error due to limited statistical power.* |  |
| 7- External influences  Given the long-time study period (2022–2024), any possible external factors should be added to the discussion. | We agree with the reviewer that, over the duration of the study, external system-level factors may have influenced lung cancer pathway performance. This issue was addressed in the revised manuscript in response to Comment 3 (causal language), where we added an explicit limitation noting that, given the pre–post design and absence of a contemporaneous control group, the influence of secular trends, unmeasured service pressures, and other external system-level factors operating over time cannot be excluded. The relevant section text is provided below. We trust that this is satisfactory.  *An additional limitation of this study relates to its pre–post design and the absence of a contemporaneous control group. As a result, it is not possible to attribute observed changes in waiting times solely to the interventions implemented, nor to exclude the influence of secular trends, unmeasured service pressures, or external system-level factors operating over the study period. Although the iterative, real-world nature of quality improvement work necessitates such designs, these findings should be interpreted as associative rather than causal, and replication within controlled or multi-centre frameworks would be required to establish causality.* | DC |
